# ﻿Morphological description and mitochondrial DNA-based phylogenetic placement of a new species of *Callistoctopus* Taki, 1964 (Cephalopoda, Octopodidae) from the southeast waters of China

**DOI:** 10.3897/zookeys.1121.86264

**Published:** 2022-09-12

**Authors:** Xiaodong Zheng, Chenxi Xu, Jiahua Li

**Affiliations:** 1 Institute of Evolution and Marine Biodiversity, Ocean University of China, Qingdao, 266003, China Ocean University of China Qingdao China; 2 Key Laboratory of Mariculture, Ocean University of China, Qingdao, 266003, China Ocean University of China Qingdao China

**Keywords:** *Callistoctopusxiaohongxu* sp. nov., COI gene, new species, octopus, taxonomy

## Abstract

In this study, we described a new species of octopus and named it *Callistoctopusxiaohongxu***sp. nov.** based on nine specimens captured in the waters of southeast China. *Callistoctopusxiaohongxu***sp. nov.** is a small to moderate-sized octopus. The most characteristic and defining morphological features are the reddish-orange to reddish-brown skin, gills with 8 or 9 lamellae per demibranch, \∧/-shaped funnel organ, and small suckers. Fragments obtained from the mitochondrial cytochrome c oxidase subunit I (COI) gene of nine specimens were 593 bp in length, and the genetic distance among the specimens of *C.xiaohongxu***sp. nov.** and the other 16 octopods ranged from 11.13 to 21.09%. Topologies resulting from ML and BI analyses of the COI gene showed a highly supported monophyletic clade (bootstrap value [BS] = 94%, posterior probability [PP] = 100%) containing all the specimens identified as *C.xiaohongxu***sp. nov.**

## ﻿Introduction

Among the cephalopods, 134 species have been recorded in the China Seas (Li 1983; Dong 1988; Zheng et al. 1999; Lu et al. 2012).Due to the influence of three strong warm currents – the Kuroshio Current (KC), the South China Sea Current (SCSC), and the Taiwan Current (TC) – water temperatures of the East China Sea and South China Sea range between 14–16 °C in coastal areas even during winter (Liu 2013), providing ideal environmental conditions to generate abundant marine biodiversity, as well as cephalopods.

Species in the genus *Callistoctopus* were previously treated as the “*Octopusmacropus* group”, from which Norman (1993) separated four new species. The current taxonomy of species in this genus is mainly based on morphological features, while there are still very limited molecular data. In Chinese waters, only two species *Callistoctopusornatus* (Gould, 1852) and *C.luteus* (Sasaki, 1929), have been recorded so far (Lu et al. 2012; Norman et al. 2014).

In this paper, we described one new species of *Callistoctopus*, which was called ‘xiaohongxu’ in Chinese for its smooth skin and reddish-brown colour, from the southeast China Sea area. The newly discovered species has been mistakenly identified and sold in fish markets of Dongshan Island in Zhangzhou, Fujian Province, as juveniles of ‘Octopus’ minor (Sasaki, 1920). However, based on the obvious differences in the size of the adult animals, gill lamellae number, and the funnel organ shape, we can readily distinguish this new species from ‘O.’ minor externally. Here we present a full morphological description and genetic analyses of the new species of octopod.

## ﻿Materials and methods

### ﻿Specimen collection

Samples were collected from Dongshan Seafood Market Pier (23°25'12"N, 117°51'0"E) in Zhangzhou, Fujian Province, China. The type specimens are deposited in the Specimen Room, Fisheries College, Ocean University of China (**OUC**), Qingdao, China. All specimens were attributed to mature or immature stages based on the absence or presence of spermatophores in males, and ovary fullness or egg development in females.

### ﻿Morphological feature analyses

All specimens were measured after being fixed according to Roper and Voss (1983) and indices were calculated on the basis of Huffard and Hochberg (2005). Abbreviations: **TL** – total length; **ML** – mantle length; **WF** – web formula (web sectors ordered from deepest to shallowest); **GC** – gill count (number of gill lamellae per outer demibranch, excluding the terminal lamella); **SC** – number of suckers on normal arms; **MWI** – mantle width index (mantle width/ML×100).; **HWI** – head width index (head width/mantle width×100); **WDI** – the web depth index (deepest web length/longest arm×100); **ALI** – arm length index (arm length/ML×100); **AWI** – arm width index (arm width/ML×100); **SDI** – sucker diameter index (sucker diameter/ML×100); **FLI** – funnel length index (funnel length/ML×100); **FFLI** – free funnel length index (free funnel length/funnel length×100); **PAI** – pallial aperture index (pallial aperture/mantle width×100); **LLI** – ligula length index (ligula length/hectocotylized arm length×100); **CaLI** – calamus length index (calamus length/ligula length×100); **HAMI** – hectocotylized arm mantle index (hectocotylized arm length/ML×100); **OAI** – opposite arm index (hectocotylized arm length/normal third arm length×100); **HASC** – number of suckers on hectocotylized arm of male; **SpC** – spermatophore count; **SpL** – spermatophore length; **SpW** – spermatophore width; **EgC** – egg count; **EgL** – egg length; **EgW** – egg width. All measurements are in millimeters and weights in grams.

The beaks and radulae were removed from the buccal mass. Then beaks were cleaned and stored in 75% ethanol. Seven beak morphological indices, upper hood length (**UHL**), upper crest length (**UCL**), upper rostrum length (**URL**), upper rostrum width (**URW**), lower hood length (**LHL**), lower crest length (**LCL**), and lower rostrum width (**LRW**), were measured to the nearest 0.01 mm by Vernier caliper (Clarke, 1986). Five ratios were calculated as follows: UHL/UCL, URW/UCL, URL/UHL, LHL/LCL, and LRW/LCL. The radulae were cleaned with 10% NaOH, air-dried, coated with gold, and then scanned using a VEGA3 scanning electron microscope. Funnel organ and anal flaps were stained with methylene blue.

### ﻿DNA extraction and sequencing

Before fixation with formalin and alcohol, about 100 mg of muscle tissue was cut from the mantle inside all chilled specimens. Total genomic DNA was extracted using a CTAB (hexadecyltrimethylammonium bromide) method (Winnepenninckx 1993). DNA was dissolved in TE buffer (10 mM Tris-HCI, 1 mM EDTA, pH 8.0) and stored at –30° C. Regions of mitochondrial cytochrome c oxidase subunit I (COI) fragments were amplified using primers referenced to *Octopusconispadiceus* Sasaki, 1917 by Ma et al. (2016). These sequences were amplified by PCR with the following conditions: 94 °C (3 min), 34 cycles of 94 °C (45 s), 50 °C (1 min), 72 °C (1 min), and a final extension of 72 °C (5 min).

### ﻿Molecular analyses

The COI sequences of the other 17 species were downloaded from GenBank (Table 1). *Vampyroteuthisinfernalis* Chun, 1903 was used as an outgroup in all analyses. ModelFinder (Kalyaanamoorthy et al. 2017) was used to select the best-fit model using the BIC criterion. Maximum likelihood phylogenies were inferred using IQ-TREE (Nguyen et al. 2015) under the GTR+I+G4+F model for 1000 ultrafast (Minh et al. 2013) bootstraps, as well as the Shimodaira-Hasegawa-like approximate likelihood-ratio test (Guindon et al. 2010). Bayesian inference phylogenies were inferred using MrBayes 3.2.6 (Ronquist et al. 2012) under GTR+I+G4+F model (4 parallel runs, 1 000 000 generations) as well, in which the initial 25% of sampled data were discarded as burn-in. And COI sequences of new species have been deposited in GenBank under accession numbers OP135961-OP135969. Pairwise comparisons of the distances based on COI gene were also calculated by MEGA X under the Kimura 2-parameter model (Kumar et al. 2018).

**Table 1.** GenBank accession numbers for species analysed in this study.

Species	GenBank numbers	References
*Amphioctopusaegina*	NC_029702	Zhang et al. (2017)
*Amphioctopusfangsiao*	HQ846126	Dai et al. (2012)
*Amphioctopusneglectus*	MH899749	Tang et al. (2019)
*Amphioctopusrex*	MF447874	Tang et al. (2019)
*Callistoctopusornatus*	HM104257	Strugnell et al. (2014)
*Callistoctopusaspilosomatis*	AB430525	Kaneko et al. (2011)
*Callistoctopusluteus*	NC_039848	Unpublished
*Callistoctopusmacropus*	MN933634	Lima et al. (2020)
*Callistoctopusxiaohongxu*	OP135961-OP135969	This study
*Cistopuschinensis*	KF017606	Cheng et al. (2013)
*Cistopustaiwanicus*	NC_023257	Cheng et al. (2013)
*Octopusvulgaris*	KU525762	Amor et al. (2017)
*Octopusbimaculatus*	NC_028547	Dominguez-Contreras et al. (2016)
*Octopusconispadiceus*	KJ789854	Ma et al. (2016)
*Octopuscyanea*	NC_039847	Unpublished
‘Octopus’ minor	HQ638215	Cheng et al. (2012)
*Octopussinensis*	MT712046	Li et al. (2021)
*Vampyroteuthisinfernalis*	NC_009689	Yokobori et al. (2007)

## ﻿Results

### ﻿Taxonomy

**Order Octopoda Leach, 1818**

#### Family Octopodidae d’Orbigny, 1840

##### ﻿Genus Callistoctopus

Taxon classificationAnimaliaOctopodaOctopodidae

Taki, 1964

395F3727-7A92-5C60-B9C9-06CF7C986F45

###### Type species.

*Callistoctopusornatus* (Gould, 1852).

##### ﻿ Callistoctopus xiaohongxu sp. nov.

Taxon classificationAnimaliaOctopodaOctopodidae

324448E1-F56B-5152-9F8F-5DCBBCBFD91A

https://zoobank.org/C4E08679-59A2-47AF-AA85-D19D1F78415B

Figs 1 , 2 , 3 , 4

###### Type material.

***Holotype***: OUC-201808200301, mature ♂, 45.5 mm ML, Dongshan Seafood Market Pier, Zhangzhou, Fujian, China, 20 August 2018, coll. ***Paratypes***: OUC-201812050301, mature ♂, 49.5 mm ML, Dongshan Seafood Market Pier, Zhangzhou, Fujian, China, 5 December 2018, coll. OUC-201812050302, mature ♂, 53.2 mm ML, Dongshan Seafood Market Pier, Zhangzhou, Fujian, China, 5 December 2018, coll. OUC-201812050303, mature ♂, 56.3 mm ML, Dongshan Seafood Market Pier, Zhangzhou, Fujian, China, 5 December 2018, coll. OUC-201806080302, immature ♀, 50.7 mm ML, Dongshan Seafood Market Pier, Zhangzhou, Fujian, China, 8 June 2018, coll. OUC-201812050305, mature ♀, 51.7 mm ML, Dongshan Seafood Market Pier, Zhangzhou, Fujian, China, 5 December 2018, coll. OUC-201812050306, mature ♀, 83.3 mm ML, Dongshan Seafood Market Pier, Zhangzhou, Fujian, China, 5 December 2018, coll.

**Figure 1.**
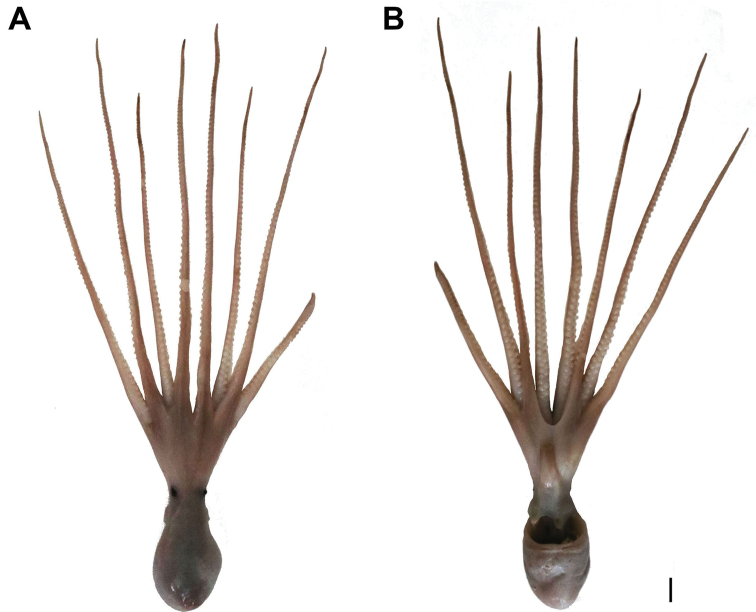
*Callistoctopusxiaohongxu* sp. nov., holotype, male, 45.5 mm ML (OUC-201808200301) **A** photograph of dorsal view **B** photograph of ventral view. Scale bars: 10 mm (**A, B**).

###### Other material.

OUC-201812050304, mature ♂, 63.2 mm ML, Dongshan Seafood Market Pier, Zhangzhou, Fujian, China, 5 December 2018, coll. OUC-201806080301, immature ♀, 41.7 mm ML, Dongshan Seafood Market Pier, Zhangzhou, Fujian, China, 8 June, 2018, coll.

###### Diagnosis.

**Figure 2.**
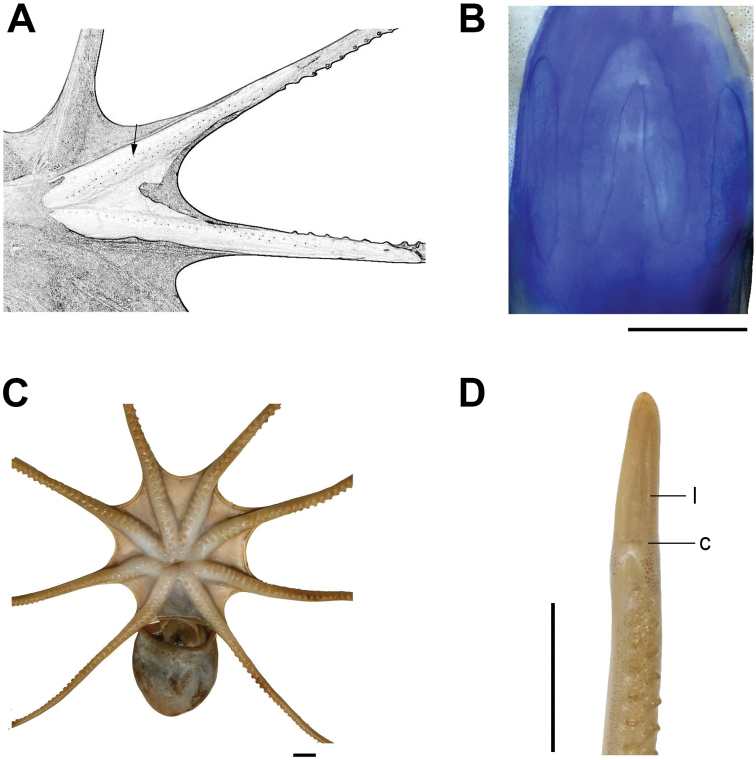
*Callistoctopusxiaohongxu* sp. nov. **A** proximal portion of arms 1–3 (left side), male, 49.5 mm ML (OUC-201812050301) **B** funnel organ, male, 53.2 mm ML (OUC-201812050302) **C** oral view of basal portion of arms, male, 63.2mm ML (OUC-201812050304) **D** distal portion of hectocotylus, male, 63.2 mm ML (OUC-201812050304). Abbreviations: c, calamus; l, ligula. Scale bars: 10 mm (**B, C, D**).

Small to moderate size (ML 41.7–83.3 mm). Colour of skin reddish-orange to reddish-brown, no papillae or patch. One or two lines of black chromatophores on the lateral margins of arms under the skin (Fig. 2A). Head narrow (HWI 23.0–39.1). Arms of moderate length (ALI 154.9–336.3), thin (AWI 8.7–18.0). Web deep (WDI 15.7–22.9). Suckers small (SDI 5.0–6.9) and biserial. Enlarged suckers absent. Funnel organ \ /\ /-shaped, long (FLI 51.0–68.5). Gills with 8–9 lamellae per demibranch. Ligula moderate size (LLI 7.0–11.6) with groove.

###### Description.

Measurements and indices of nine specimens are presented in Table 2. Small to moderate-size species (ML 41.7–83.3 mm), total length (TL) 195.7–382.1 mm, body weight up to 39.2 g. Skin smooth, one or two lines of black chromatophores on the lateral margins of arms under the skin (Fig. 2A). Mantle slightly ovoid to elongate, muscular. Head width narrower than mantle width (HWI 23.0–39.1). Stylets absent. Funnel long (FLI 51.0–68.5), free funnel length around 24–46% funnel length (FFLI 23.9–46.0), funnel organ \∧/-shaped (Fig. 2B). Outer limbs slightly shorter than medial limbs. Arms moderate length (ALI 154.9–336.3), slender (AWI 8.7–18.0), dorsal arms always longest (arm formula of most specimens belongs to 1 > 2 > 4 > 3). Suckers in two rows (Fig. 2C), small (SDI 5.0–6.9). In larger animals, 157–198 suckers on each normal arm, and the first or second arm has the most suckers. Enlarged suckers absent. Webs deep (WDI 15.7–22.9), typical web formula A > B > C > D > E. The third right arm of mature males hectocotylized, length approximately 60–80% of the opposite arm (Fig. 2D). Ligula of moderate size, robust and cylindrical with deep groove. LLI ranges from 7.0–11.6 of arm length. Calamus of moderate size, around 25–30% of ligula length (CaLI 26.3–31.6). Hectocotylized arm with 70–83 suckers. Gills with 8–9 lamellae per demibranch.

**Table 2.** Measurements (mm) and indices for *Callistoctopusxiaohongxu* sp. nov. Abbreviation: D, damaged.

Name	OUC-201808200301	OUC-201812050301	OUC-201812050302	OUC-201812050303	OUC-201812050304	OUC-201806080301	OUC-201806080302	OUC-201812050305	OUC-201812050306
Status	Holotype	Paratype	Paratype	Paratype			Paratype	Paratype	Paratype
Sex	♂	♂	♂	♂	♂	♀	♀	♀	♀
Maturity	mature	mature	mature	mature	mature	immature	immature	mature	mature
TL	212.7	216.3	212.1	258.6	283.9	195.7	208.6	234.8	382.1
TW(g)	30.6	24.5	37.3	28.8	23.4	17.6	16.1	25.1	39.2
ML	45.5	49.5	53.2	56.3	63.2	41.7	50.7	51.7	83.3
MWI	72.5	69.7	65.6	66.2	65.0	64.5	60.5	77.4	46.2
HWI	35.2	29.1	23.9	30.7	27.2	30.0	27.6	39.1	23.0
PAI	120.9	116.6	111.5	116.5	103.2	149.6	111.0	125.0	98.3
FLI	63.1	65.1	66.2	67.1	53.2	61.4	57.8	68.5	51.0
FFLI	46.0	35.1	23.9	26.2	25.9	40.6	32.4	34.2	33.6
WDI	17.0	17.3	22.9	15.7	18.3	19.8	20.0	16.9	16.2
AL1I	333.8	331.9	237.2	322.7	196.7	D	285.4	324.8	294.5
AL2I	336.3	276.0	241.2	302.1	304.1	322.5	251.1	313.5	154.9
AL3I	291.9	273.1	231.8	220.1	220.4	294.5	226.8	272.7	293.0
AL4I	296.7	259.2	193.2	265.2	229.9	299.0	211.8	281.8	237.1
AWI	15.2	13.8	14.3	13.2	11.6	14.5	18.0	16.2	8.7
LLI	7.0	11.6	9.8	9.6	10.4	–	–	–	–
CaLI	31.6	26.3	28.2	30.9	31.1	–	–	–	–
HAMI	184.2	160.8	162.4	182.8	155.5	–	–	–	–
OAI	63.1	58.9	70.1	81.8	70.6	–	–	–	–
SDI	6.9	6.5	5.7	6.6	6.5	5.4	5.0	5.9	5.4
HASC	79	82	83	83	70	–	–	–	–
SC	157	191	171	198	195	191	163	178	177
GC	8	9	8	9	8	8	8	8	8
SpC	–	6	8	–	4	–	–	–	–
SpL	–	37.8	58.1	–	79.1	–	–	–	–
SpW	–	1.4	1.5	–	1.6	–	–	–	–
EgC	–	–	–	–	–	–	–	64	67
EgL	–	–	–	–	–	–	–	14.0	14.5
EgW	–	–	–	–	–	–	–	4.3	3.3
UHL/UCL	–	0.31	0.28	0.28	0.29	–	–	0.32	0.28
URW/UCL	–	0.18	0.13	0.17	0.15	–	–	0.16	0.12
URL/UHL	–	0.33	0.28	0.38	0.23	–	–	0.27	0.23
LHL/LCL	–	0.38	0.39	0.40	0.45	–	–	0.34	0.36
LRW/LCL	–	0.33	0.32	0.41	0.36	–	–	0.26	0.30

**Figure 3.**
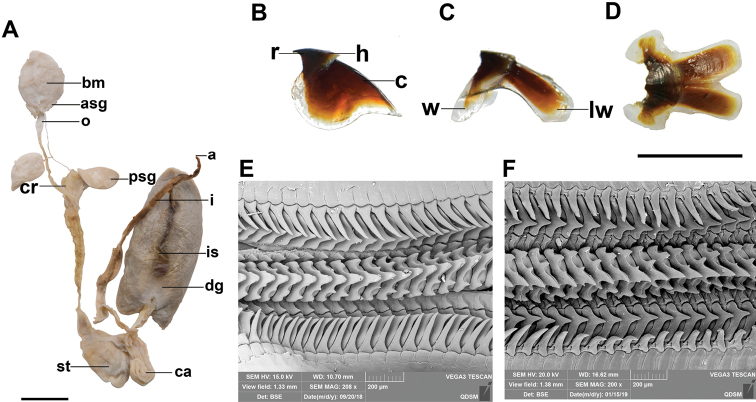
*Callistoctopusxiaohongxu* sp. nov. **A** digestive system, female, 83.3 mm ML (OUC-201812050306) **B** upper beak, lateral view, female, 50.7 mm ML (OUC-201806080302) **C** lower beak, lateral view, female, 50.7 mm ML (OUC-201806080302) **D** lower beak, ventral view, female, 50.7 mm ML (OUC-201806080302) **E, F** scanning electron micrograph of radulae, male, 53.2 mm ML (OUC-201812050302). Abbreviations: a, anus; asg, anterior salivary gland; bm, buccal mass; c, crest; ca, caecum; cr, crop; dg, digestive gland; h, hood; i, intestine; is, ink sac; lw, lateral wing; o, oesophagus; psg, posterior salivary gland; r, rostrum; st, stomach; w, wing. Scale bars: 10 mm (**A–D**); 200 μm (**E, F**).

Digestive tract (Fig. 3A). Anterior salivary glands small, approximately one-third length of buccal mass. Posterior salivary glands triangular and smaller than buccal mass. Oesophagus long. Spiral caecum with one whorl. Intestine long. Digestive gland well developed, brown. Ink sac present, embedded in the digestive gland and attached to the intestine posteriorly. Ink sac opening into the anus. Anal flaps small.

Upper beak (Fig. 3B) with short rostrum, narrow hood, and slightly curved crest. Ratios of upper beak measurements 0.28–0.32 for UHL/UCL, 0.12–0.18 for URW/UCL, and 0.23–0.38 for URL/UHL. Lower beak (Fig. 3C, D) with a blunt rostrum, narrow hood, moderately broad wings and flared lateral walls separated in posterior, posterior notch deep. Radula (Fig. 3E, F) with 7 teeth and 2 marginal plates per transverse row. Ratios of lower beak measurements 0.34–0.45 for LHL/LCL and 0.26–0.41 for LRW/LCL. Rhachidian tooth with 1–2 lateral cusps on each side; first lateral teeth small, sharp; second lateral teeth broad-based triangular, larger than first, sharp; marginal teeth long, curved, sharply pointed, longer than second lateral teeth; marginal plates flat.

**Figure 4.**
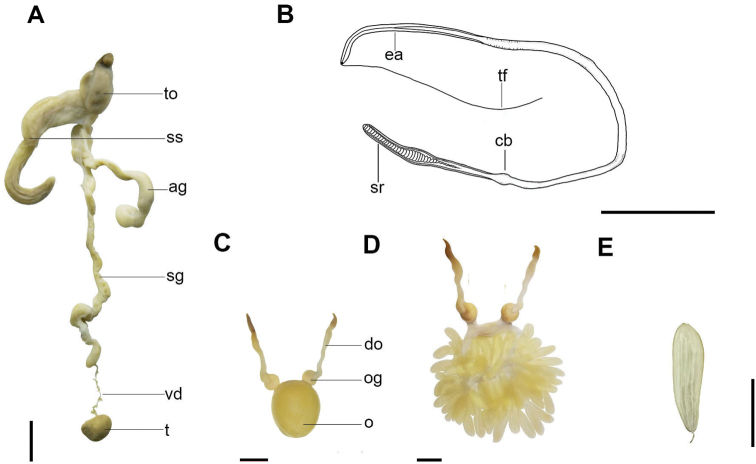
*Callistoctopusxiaohongxu* sp. nov **A** reproductive system of male, 56.3 mm ML (OUC-201812050303) **B** spermatophore, male, 56.3 mm ML (OUC-201812050303) mm **C** reproductive system of female, 51.7 mm ML (OUC-201812050305) **D** egg cluster, female, 51.7 mm ML (OUC-201812050305) **E** single egg female, 51.7 mm ML (OUC-201812050305). Abbreviations: ag, accessory gland; cb, cement body; do, distal oviduct; ea, ejaculatory apparatus; o, ovary; og, oviducal gland; sg, spermatophore gland; sr, sperm reservoir; ss, spermatophore storage sac; t, testis; tf, terminal filament; to, terminal organ; vd, vas deferens. Scale bars: 10 mm.

Male reproductive tract (Fig. 4A). In mature males, the terminal organ inverse 6-shaped. Spermatophore storage sac long. Accessory gland curved, longer than spermatophore storage sac. Spermatophore gland long. Vas deferens very short, narrow. Testis roundish, small. Spermatophores (Fig. 4B) of moderate size, average length 60 mm, approximately 75% ML, narrow (average 1.5 mm in width); approximately 4–8 spermatophores in storage sac.

Female reproductive tract (Fig. 4C–E). Ovary large, round in mature females. Two distal oviducts long. Two oviducal glands wider than distal oviducts. Mature females with approximately 65 large eggs (average 14.3 mm in length).

**Figure 5.**
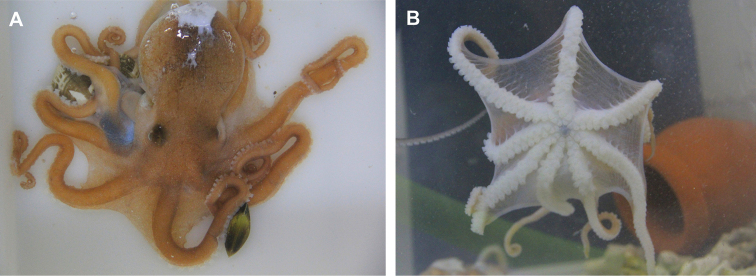
*Callistoctopusxiaohongxu* sp. nov. **A** live specimen **B** net-like structure on web.

Integument (Fig. 5A). Colour of live animal reddish-orange. Animal turning white when stressed or post mortem. In live animals, a linear structure appears on the tissue connecting two adjacent arms, forming a net-like structure (Fig. 5B). Arm chromatophores under the skin distinct.

###### Etymology.

The name ‘*xiaohongxu*’, which refers to its small body size and reddish body colour, is the phonetic translation of the local Chinese name of this species in Zhangzhou, where specimens were collected.

###### Distribution.

According to fishermen in Zhangzhou, this species is distributed in the East China Sea and the South China Sea, mainly in Quanzhou, Fujian Province to Shanwei, Guangdong Province.

###### Molecular analyses.

**Figure 6.**
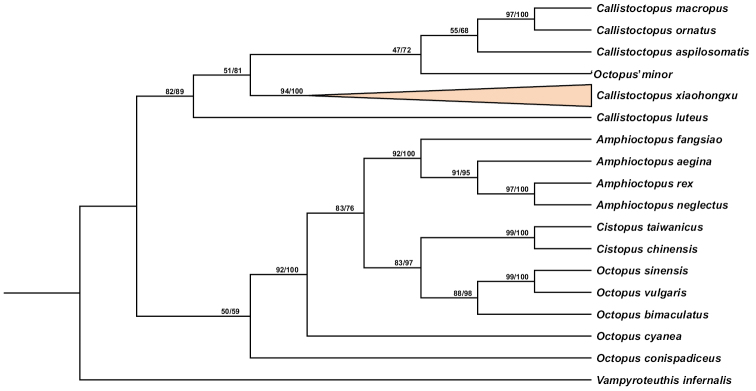
Phylogenetic trees derived from Maximum likelihood (ML) and Bayesian inference (BI) methods based on the *COI* gene. Numbers at each node are bootstrap (left) for ML and posterior probability (right) for BI analyses, respectively.

Phylogenetic analyses were performed based on the fragments of the COI gene using Maximum likelihood (ML) and Bayesian inference (BI) methods. Fragments 593 bp in length were obtained from the mitochondrial COI gene of nine specimens. Both ML and BI trees showed a similar topology (Fig. 6) with a highly supported monophyletic clade (bootstrap value [BS] = 94%, posterior probability [PP] = 100%) containing all nine specimens identified as *Callistoctopusxiaohongxu* sp. nov. *C.xiaohongxu* sp. nov. belonged to the clade of ‘*O*’. *minor* and other four species of *Callistoctopus* with [BS] = 82% and [PP] = 89%, respectively. Moreover, the COI gene analyses suggested that species of the genus *Octopus* used in this study were not clustered into one clade. Additionally, the genetic distance of *C.xiaohongxu* sp. nov. and the other 16 Octopodidae species ranged from 11.13 to 21.09% (Table 3).

**Table 3.** Pairwise comparison of the genetic distances among Octopodidae species based on the COI gene. Abbreviations: *A.a.*, *Amphioctopusaegina*; *A.f.*, *Amphioctopusfangsiao*; *A.n.*, *Amphioctopusneglectus*; *A.r.*, *Amphioctopusrex*; *Ca.a.*, *Callistoctopusaspilosomatis*; *Ca.l.*, *Callistoctopusluteus*; *Ca.m.*, *Callistoctopusmacropus*, *Ca.o.*, *Callistoctopusornatus*; *Ca.x., Callistoctopusxiaohongxu*; *Ci.c.*, *Cistopuschinensis*; *Ci.t.*, *Cistopustaiwanicus*; *O.b.*, *Octopusbimaculatus*; *O.co.*, *Octopusconispadiceus*; *O.cy.*, *Octopuscyanea*; ‘*O.’ m.*, ‘Octopus’ minor; *O.s.*, *Octopussinensis*; and *O.v.*, *Octopusvulgaris*.

	*A.a.*	*A.f.*	*A.n.*	*A.r.*	*Ca.a.*	*Ca.l.*	*Ca.m.*	*Ca.o.*	*Ca.x.*	*Ci.c.*	*Ci.t.*	*O.b.*	*O.co.*	*O.cy.*	‘*O.’ m.*	*O.s.*
*A.a.*	–															
*A.f.*	14.29	–														
*A.n.*	12.43	16.36	–													
*A.r.*	11.78	16.56	11.03	–												
*Ca.a.*	20.32	20.34	22.76	21.09	–											
*Ca.l.*	20.10	19.02	20.18	19.50	14.54	–										
*Ca.m.*	19.62	20.57	21.27	20.37	12.22	15.12	–									
*Ca.o.*	21.03	20.38	19.63	21.13	10.95	15.40	5.01	–								
*Ca.x.*	18.51	19.40	20.29	18.63	14.04	15.54	12.18	11.97	–							
*Ci.c.*	18.26	18.81	20.97	19.47	21.40	20.55	19.70	20.22	17.76	–						
*Ci.t.*	16.45	19.01	16.93	18.05	19.87	21.11	18.49	17.58	19.08	13.11	–					
*O.b.*	18.05	14.74	20.21	20.68	20.40	19.27	19.96	20.50	17.40	16.28	19.04	–				
*O.co.*	20.09	19.22	18.73	20.36	16.94	17.68	19.02	17.39	16.63	20.87	20.34	21.17	–			
*O.cy.*	14.95	16.74	17.47	18.61	17.19	17.65	17.83	17.16	15.33	17.17	16.24	15.00	18.05	–		
‘*O’. m*	19.87	19.44	23.52	22.04	13.50	14.17	10.77	11.43	11.13	18.28	20.60	19.21	19.01	17.59	–	
*O.s.*	14.30	17.90	17.47	18.37	21.07	20.44	20.35	20.36	20.72	17.68	17.93	15.27	20.36	16.99	20.81	–
*O.v.*	14.37	17.51	17.23	18.29	21.51	21.04	20.14	20.15	21.09	18.87	18.60	15.21	20.69	18.30	20.69	2.97

## ﻿Discussion

As mentioned previously, *Callistoctopusxiaohongxu* sp. nov. has been mistakenly identified and sold in fish markets as the juveniles of ‘*O*’. *minor*, because they are similar in having smooth skin and reddish-brown colour in chilled specimens. However, *C.xiaohongxu* sp. nov. and ‘*O*’. *minor* can be readily distinguished by the morphological characteristics compared in Table 4. *Callistoctopusxiaohongxu* sp. nov. has:

**Table 4.** Comparison of key morphological characters between *Callistoctopusxiaohongxu* sp. nov., *Callistoctopus* species, and ‘Octopus’ minor Sasaki, 1920.

Item	*Callistoctopusxiaohongxu* sp. nov.	*Callistoctopus* species	‘*Octopus’ minor* Sasaki, 1920
Data source	this study	Norman et al. 2014	Norman et al. 2014
Colour	reddish-orange to reddish-brown, no spot	Typically, red-brown to red, white spots or bars on mantle, head and arms	red-brown, light yellow spots on mantle surface
Sculpture	smooth	smooth or with scattered low papillae	smooth
GC	8–9	10–14	10–12
Funnel organ	\ /\ /-shaped	W, UU or VV-shaped	V V-shaped
WDI	15.7 to 22.9	around 7 to 28	deepest around 10
ALI	154.9–336.3	300–800	400–500
OAI	58.9–81.8	around 40–95	around 50
Enlarged suckers	absent	absent	present
Ligula	cylindrical with groove, LLI 7.0–11.6	cylindrical with deep groove, LLI around 1.5–9	spoon-like with wide hollow groove, LLI around 18–23

1. no spots on mantle surfaces (‘ *O*’. *minor* has light yellow spots);
2. a \∧/-shaped funnel organ (the funnel organ shape of ‘ *O*’. *minor* is V V-shaped);
3. gills with 8 or 9 lamellae per demibranch (10–12 lamellae per demibranch in ‘ *O*’. *minor*);
4. no enlarged suckers in mature males (‘ *O*’. *minor* has enlarged suckers);
5. cylindrical ligula with groove (spoon-like with a wide hollow groove in ‘ *O*’. *minor*).

*Callistoctopusxiaohongxu* sp. nov. is also distinct from other species of *Callistoctopus* (Table 4). Compared to the key morphological characters, *C.xiaohongxu* sp. nov. has no spot on the skin (vs other *Callistoctopus* species that have white spots or bars on the mantle, head, and arms), fewer gill lamellae (gill lamellae 8–9 vs 10–14 in *Callistoctopus* species), funnel organ \ /\ /-shaped (vs W, UU or VV-shaped in other species), and relatively shorter arms (ALI 154.9–336.3 vs 300–800 in the other *Callistoctopus* species).

Judging from the K2P genetic distance (Table 3), *C.xiaohongxu* sp. nov. can be separated from the other 16 species of Octopodidae by genetic distances ranging from 11.13 to 21.09%. According to the phylogenetic tree (Fig. 6), *C.xiaohongxu* sp. nov. has a close relationship to ‘*O*’. *minor* and four species of *Callistoctopus* with [BS] = 82% and [PP] = 89%. However, the taxonomic status of ‘*O*’. *minor* is unresolved, and it is placed in the genus *Octopus* provisionally. Besides, the very limited suite of molecular data suggested that genetic relationships among species of the genus *Callistoctopus* need further studies. Still, for the accurate phylogenetic status of ‘*O*’. *minor*, more research would be required to establish the relationships among species of *Octopus* and *Callistoctopus*.

*Octopus* is one of the most species-rich cephalopod genera but was considered a ‘catch-all’ genus by Guzik et al. (2005). It is not monophyletic in its current composition and needs revision and robust phylogenetic analyses (Strugnell et al. 2005; Dai et al. 2012; Amor et al. 2015; Ritschard et al. 2019; Tang et al. 2020). In our study, species in *Octopus* were not clustered into one clade. Accordingly, our study supports the polyphyly of the genus *Octopus*.

We are planning to analyse the mitochondrial genome of *C.xiaohongxu* sp. nov. in the future. Better taxon sampling would facilitate a better understanding of octopod phylogeny as well as a better substantiated generic assignment of *C.xiaohongxu* sp. nov.

## Supplementary Material

XML Treatment for Callistoctopus

XML Treatment for Callistoctopus xiaohongxu
